# Small bowel capsule endoscopy examination and open access database with artificial intelligence: The SEE‐artificial intelligence project

**DOI:** 10.1002/deo2.258

**Published:** 2023-06-22

**Authors:** Akihito Yokote, Junji Umeno, Keisuke Kawasaki, Shin Fujioka, Yuta Fuyuno, Yuichi Matsuno, Yuichiro Yoshida, Noriyuki Imazu, Satoshi Miyazono, Tomohiko Moriyama, Takanari Kitazono, Takehiro Torisu

**Affiliations:** ^1^ Department of Medicine and Clinical Science Graduate School of Medical Science Kyushu University Fukuoka Japan; ^2^ Department of Endoscopic Diagnostics and Therapeutics Kyushu University Hospital Fukuoka Japan; ^3^ International Medical Department Kyushu University Hospital Fukuoka Japan

**Keywords:** artificial intelligence, capsule endoscopy, diagnostic imaging, gastrointestinal tract, intestine small

## Abstract

**Objectives:**

Artificial intelligence (AI) may be practical for image classification of small bowel capsule endoscopy (CE). However, creating a functional AI model is challenging. We attempted to create a dataset and an object detection CE AI model to explore modeling problems to assist in reading small bowel CE.

**Methods:**

We extracted 18,481 images from 523 small bowel CE procedures performed at Kyushu University Hospital from September 2014 to June 2021. We annotated 12,320 images with 23,033 disease lesions, combined them with 6161 normal images as the dataset, and examined the characteristics. Based on the dataset, we created an object detection AI model using YOLO v5 and we tested validation.

**Results:**

We annotated the dataset with 12 types of annotations, and multiple annotation types were observed in the same image. We test validated our AI model with 1396 images, and sensitivity for all 12 types of annotations was about 91%, with 1375 true positives, 659 false positives, and 120 false negatives detected. The highest sensitivity for individual annotations was 97%, and the highest area under the receiver operating characteristic curve was 0.98, but the quality of detection varied depending on the specific annotation.

**Conclusions:**

Object detection AI model in small bowel CE using YOLO v5 may provide effective and easy‐to‐understand reading assistance. In this SEE‐AI project, we open our dataset, the weights of the AI model, and a demonstration to experience our AI. We look forward to further improving the AI model in the future.

## INTRODUCTION

The small bowel is a 5–6 m long organ located between the stomach and the colon, making it difficult to observe directly. Since the initial report of capsule endoscopy (CE) in 2000,^1^ the small bowel has increasingly been screened using CE rather than X‐ray radiography.^2^ Current guidelines recommend prompt CE for patients with obscure gastrointestinal bleeding and those with Crohn's disease or celiac disease who have not yet received a confirmed diagnosis.^3^ CE of the small bowel involves the acquisition of two to six frames per second for 2 to 8 hours, resulting in tens of thousands of frames being output as a video during a single examination. Clinicians require approximately one hour to view and interpret this video, which is more labor‐intensive and expensive than a standard upper gastrointestinal endoscopy.^4^ Automated reading by artificial intelligence (AI) is currently the focus of research due to the significant effort required in CE and the risk of missing disease lesions.^5^ In medicine, AI models have demonstrated considerable progress in detecting important findings in head computed tomography,^6^ skin cancer,^7^ and diabetic retinopathy,^8^ as well as lung computed tomography images in patients with coronavirus disease.^9^ In the field of lower gastrointestinal endoscopy, AI models have aided in the detection of colorectal polyps,^10^ and some of these models are currently used in clinical practice.

Previously, reading aids in small bowel CE have been explored for the detection of bleeding through image coloration^11^ and the use of support vector machines.^12^ Techniques like support vector machines were machine learning methods. Nevertheless, conventional machine learning methods have encountered difficulties in selecting and extracting two‐dimensional image recognition features. Convolutional neural network emerged as a deep learning model that automatically extracts required feature vectors from the training data.^13^ While these techniques are rooted in machine learning, the introduction of convolutional neural network has profoundly impacted the field of image recognition technology. In 2012, AlexNet, which utilizes convolutional neural network, was used in an object image recognition competition (the ImageNet Large Scale Visual Recognition Challenge) to classify 14 million images into 1000 classes.^13^ Since then, many AI models have been demonstrated to be effective in clinical practice for image classification,^14^, ^15^ including the detection of Gastrointestinal bleeding,^16^ protrusion,^17^ and inflammatory bowel disease.^18^ However, small bowel CE images often depict multiple disease lesions in a single shot, and it is challenging to create a practical model based on simple image classification alone. An object detection model that indicates the location of disease lesions in an image would be appropriate for the physician to understand, although the method to generate such a model is complex. As far as we know, AI models alone to identify multiple types of clinically necessary lesions from CE images with a high level of expertise have not been achieved. In this paper, we analyzed a large dataset and created an AI model, aiming to explore relevant modeling problems.

## METHODS

### Data extraction and annotation

**FIGURE 1:**
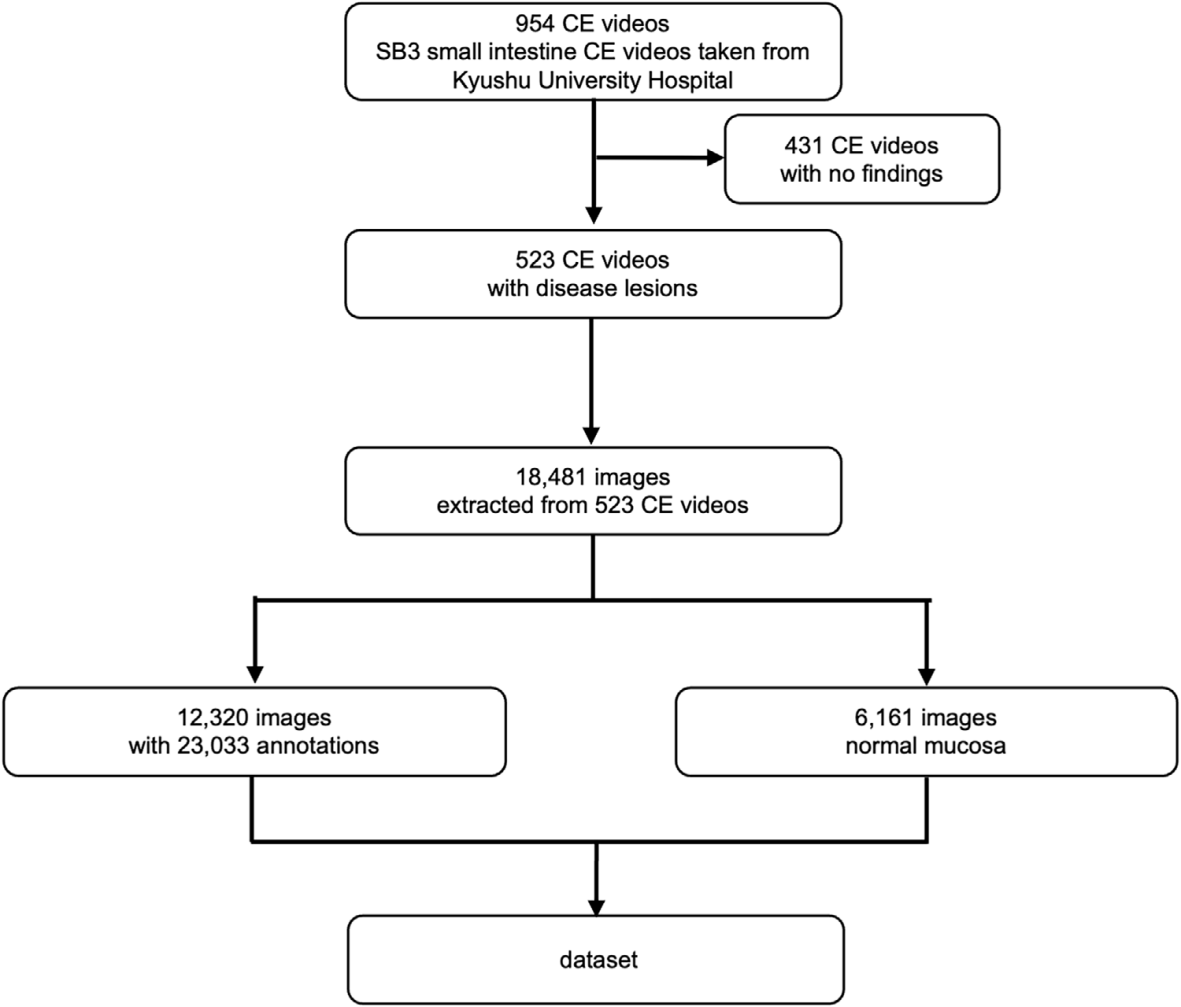
Image extraction procedure of the dataset. We used 523 of 954 collected capsule endoscopy (CE) videos as the dataset. This dataset contains 23,033 annotation labels.

In the Small Bowel CE Examination with Object Detection AI Model (The SEE‐AI) project, we retrospectively analyzed anonymized videos from 954 patients who underwent small bowel CE (PillCam SB 3; Medtronic, Minneapolis, MN, USA) at Kyushu University Hospital from September 2014 to June 2021. Participants were given the opportunity to opt out of the study, which was approved by the Ethics Review Committee of Kyushu University (“Approval No. 2021–213”). Individual consent forms were not required because this was a retrospective study with anonymized data by the Ethics Committee. All included patients fasted for at least 12 h before undergoing small bowel CE. Most examinations were performed while the patients were hospitalized. Physicians with 3–5 years of clinical experience initially reviewed the medical reports, which were then reviewed by specialists with more than 7 years of experience to identify clinically important images. Image extraction was performed by RAPID for PillCam (Medtronic), a dedicated analysis software for the PillCam SB3 endoscopy system, which extracted 576‐pixel x 576‐pixel JPEG images from the captured videos. Because some patients had no disease lesions, images were ultimately extracted from 523 of 954 videos. For anonymization, each image's area outside the frame (containing personal data) was blackened. The dataset includes 18,481 images, 12,320 of which contain disease lesions and 6161 of which are normal mucosal images with various mucosal backgrounds. Figure 1 shows the extraction procedure, and Table 1 shows the disease backgrounds of patients in the dataset. All images were annotated by seven specialists using VOTT ver1.0.8 (Microsoft, Washington, WA, USA), with square bounding boxes and 12 annotation labels deemed clinically useful. All annotation labels were checked and any discrepancies were resolved through discussion among the specialists. The quantities of food residue and air bubbles in the gastrointestinal tract varied among individuals. However, no selection was based on image quality as long as the disease lesions were discernible. In anticipation of future data being utilized by the scientific community, on our public dataset we did not crop, deform, or change the brightness or saturation of the images. As a point of note, in YOLOv5, data augmentation can be used as an internal processing step, and we have employed standard augmentation techniques in creating our models.

**TABLE 1:** Disease backgrounds included in the dataset *(n* = 523).

Characteristics		
No. of images		18,481
	With disease lesions	12,320
	No. of annotations	23,033
**Cause**		
	Follicular lymphoma	77
	Crohn's disease	58
	Angiodysplasia	39
	MALT lymphoma	22
	Familial adenomatous polyposis	23
	Diffuse large B‐cell lymphoma	17
	Cowden syndrome	17
	Peutz‐Jeghers syndrome	14
	NSAIDs ulcer	9
	GIST, adenoma	7
	Amyloidosis	6
	IgA vasculitis, anastomotic ulcer	3
	Small intestine cancer, Bechet's disease, intestinal tuberculosis, Protein‐losing gastroenteropathy, metastatic cancer, Arteriovenous malformation, adult T‐cell leukemia‐lymphoma, Eosinophil enteritis, chronic enteropathy associated with SLCO2A1	2
	Radioactive enteritis, ischemic enteritis, T‐cell lymphoma, Plasma cell tumor, Cryoglobulinemia, Intestinal emphysema, Polyarteritis nodosa, Scleroderma, Cavernous hemangioma, Olmesartan Associated Enteropathy, Cancer of unknown primary, Celiac disease, Sarcoma, Giardia, Ulcerative Colitis, Systemic lupus erythematosus, Neuroendocrine neoplasm, Cytomegalovirus enteritis	1
	Unknown disease name	196

Disease backgrounds of collected images in the dataset. MALT; mucosa associated lymphoid tissue, NSAIDs; non‐steroidal anti inflammatory drug, GIST; gastrointestinal stromal tumor.

To classify various abnormal lesions of the small bowel without any omission, we proposed 12 types of annotations. The specific annotation definitions were as follows:

- Angiodysplasia: areas of erythema with suspected capillary lesions
- Erosion: areas of mucosal damage such as erosions, ulcers, and notches
- Stenosis: areas of constriction and rigidity
- Lymphangiectasis: areas containing lymphatic vessels larger than a point
- Lymph follicle: areas containing normal follicles and suspected lymphatic follicles
- Submucosal tumor: areas resembling submucosal tumors
- Polyp‐like: elevated lesions with a base or areas of suspected adenoma
- Bleeding: areas of apparent hemorrhage, exclude bile‐colored intestinal fluid
- Diverticula: areas of the suspected diverticulum
- Redness: areas of redness and edema that may be related to inflammation
- Foreign body: foreign objects other than food
- Venous: areas with venous structures

### AI modeling and test validation

We tested the technical quality of the dataset using YOLO v5^19^ to evaluate the efficacy and limitations of our object detection AI model and annotation system. YOLO v5 is an open‐source object detection algorithm developed by Glenn Jocher (Ultralytics, Los Angeles, CA, USA) and released in June 2020. We can easily use YOLO v5 in Google Colaboratory. We trained YOLO v5x with larger parameters to train an object detection model using the dataset. The AI model was created using 17,085 of the 18,481 images in the dataset, comprising 11,043 abnormal images and 6,042 normal mucosa images with 20,629 annotated labels. The model was generated on a data center GPU (Tesla V100 SXM2 16 GB; NVIDIA, Santa Clara, CA, USA) and required over 40 hours of computation time. The starting weights for model creation were pre‐trained on COCO128,^20^ with a batch size of eight. Yolov5 reads images through hyperparameters determining the augmentations to each image. The model's mean Average Precision becomes constant during the latter half of 200 epochs, and the model is stopped being created at 300 epochs to prevent overfitting. Assuming that each augmentation produces a unique augmented image, the total number of images employed for training is 18,481×300. We employed three‐fold cross‐validation. Intersection over union measures the overlap between the AI‐detected region and the truth annotation area. In our AI validation, an intersection over a union value >0.5 was considered indicative of a correct answer. We used precision, recall, and F1‐score as model performance indicators during AI creation, which is commonly used in object detection. The indicators are defined as follows.

Precision (i.e., positive predictive value)

The proportion of samples that test positive, calculated as true positives divided by the sum of true positives and false positives.

Recall (i.e., sensitivity, probability of detection, or true positive rate)

The proportion of positive samples that correctly test positive out of all positive samples, calculated as true positives divided by the sum of true positives and false negatives.

F1‐score

A measure of test accuracy, calculated as the harmonic mean of precision and recall using the formula; 2 x precision x recall divided by the sum of precision and recall.

We utilized the residual 1396 images from the dataset for test validation. This validation of the test was executed to evaluate the performance of the AI using data that was not employed during the AI's construction. The calculation of the receiver operating characteristic (ROC) curve and other metrics such as true positives, true negatives, false positives, false negatives, sensitivity, and specificity were determined by analyzing the annotations per frame in 1396 test‐validated images. When multiple annotations of the same type are present in a single image, the bounding box with the highest confidence rate is utilized for the calculation. The cut‐off value was determined based on the Youden Index, calculated by adding sensitivity and specificity while subtracting one. Typically, the cut‐off value is set at the highest point of the Youden Index.

## RESULTS

**FIGURE 2:**
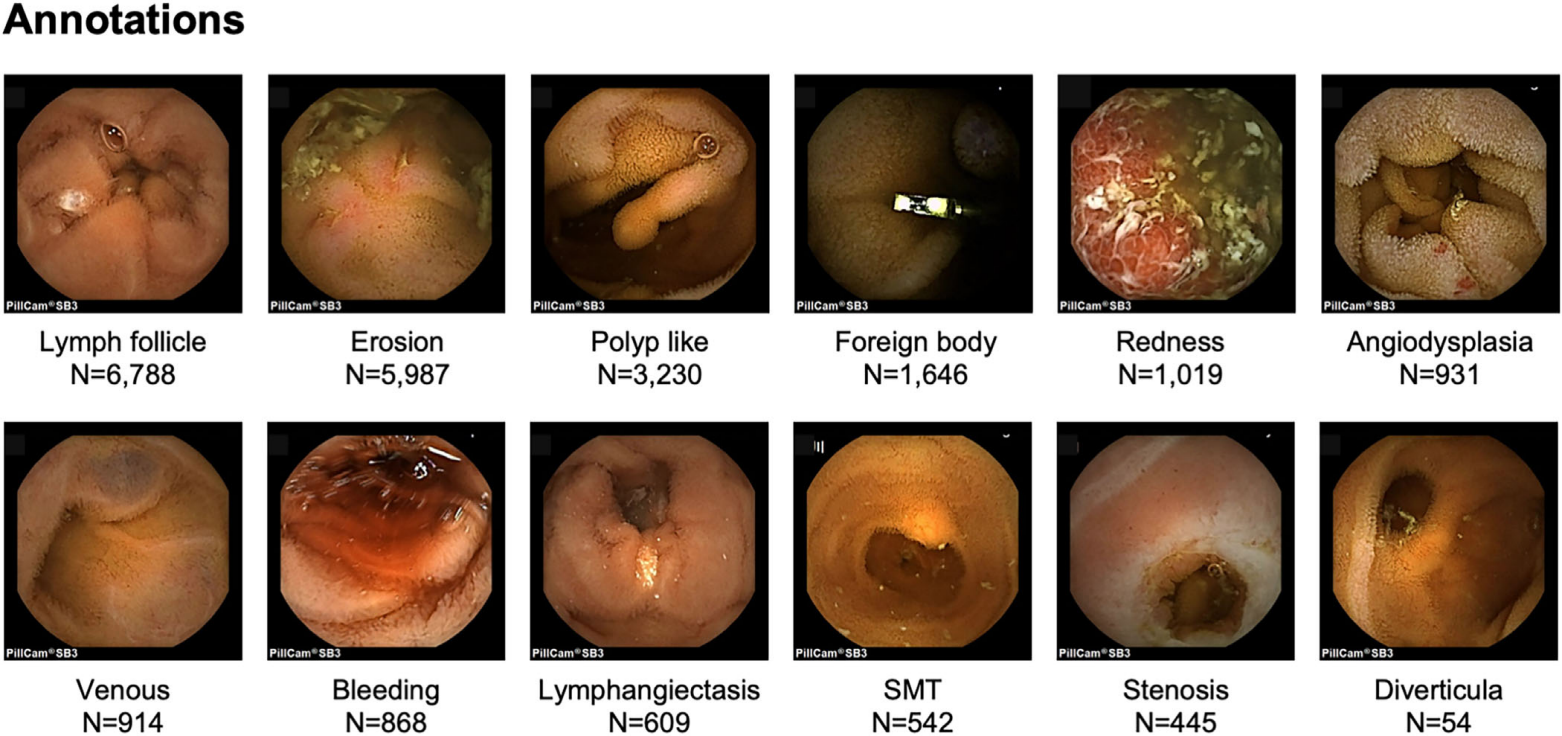
Type of annotations in the dataset. Representative images of the annotations and the distribution of annotation label numbers in the dataset; the number of labels per annotation ranged from 54 to 6788. The specific annotation definitions were as follows: Angiodysplasia; areas of erythema with suspected capillary lesions, Erosion; areas of mucosal damage such as erosions, ulcers, and notches, Stenosis; areas of constriction and rigidity, Lymphangiectasis; areas containing lymphatic vessels larger than a point, Lymph follicle; areas containing normal follicles and suspected lymphatic follicles, Submucosal tumor (SMT); areas resembling submucosal tumors, Polyp like; elevated lesions with a base or areas of suspected adenoma, Bleeding; areas of apparent hemorrhage, exclude sites that have become darker and are distant from the source of bleeding, Diverticula; areas of the suspected diverticulum, Redness; areas of redness and edema that may be related to inflammation, Foreign body; foreign objects other than food, Venous; areas with venous structures.

**FIGURE 3:**
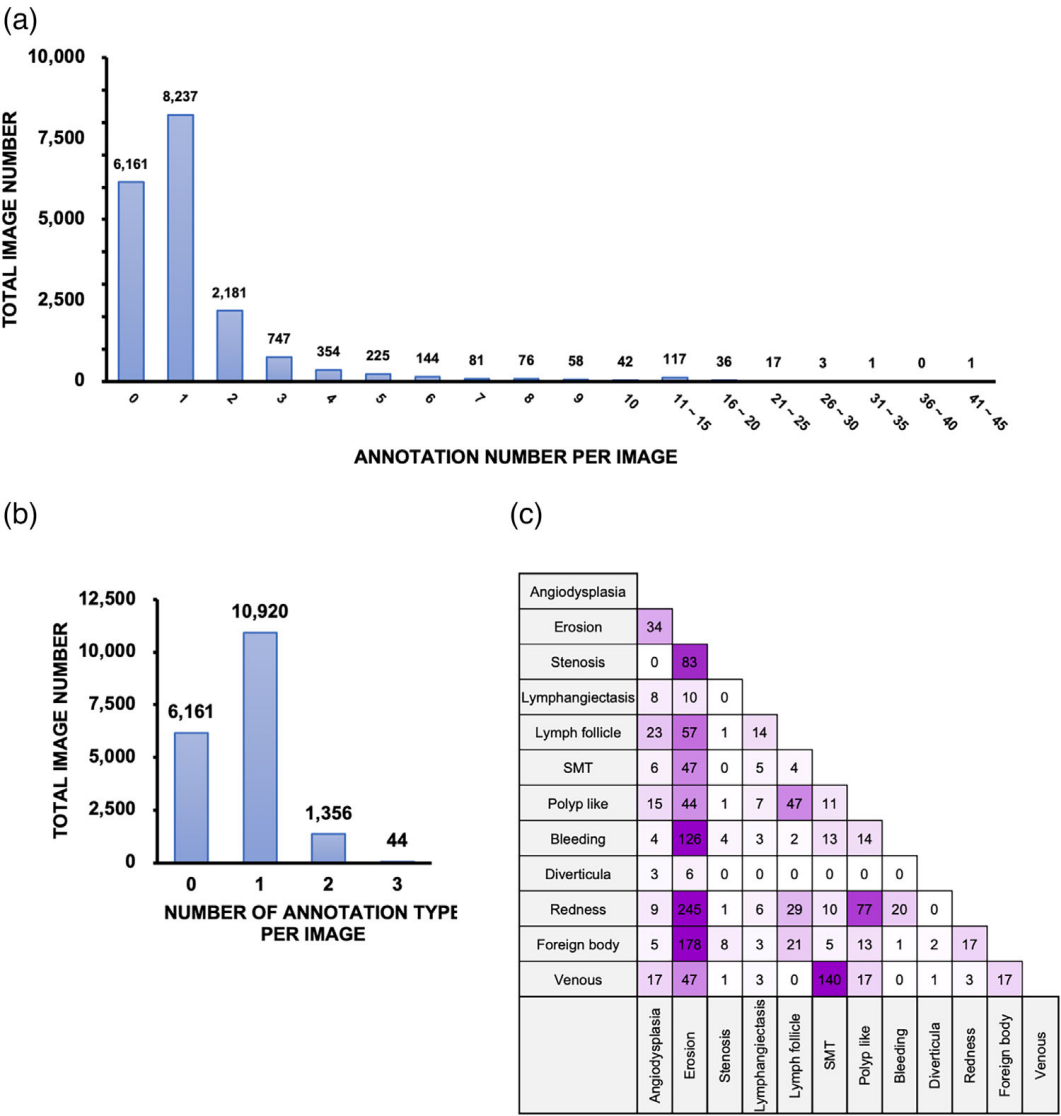
Features of annotations in the dataset. (a) Distribution of annotation labels per image and 4083 of 18,481 images have multiple lesions in one image. Approximately 2% of all images contained more than 10 annotation labels. (b) Distribution of annotation label types per image. There are two different abnormalities in 1356 images and three types in 44 images. (c) The number of annotation labels appears in the same image; for example, “erosion” and “redness” both tended to appear more often with other annotation label types.

Figure 2 contains representative images of the annotations and shows the distribution of annotation labels in the dataset. Figure 3a shows how many abnormal lesions were detected in one image, and 4083 images out of 18,481 images have multiple disease lesions. Figure 3b shows that some images have different types of annotations in one image. There are two different abnormalities in 1356 images and three types in 44 images. The combination of annotations in the same image is shown in Figure 3c. These results indicate that images of diffuse lesions containing multiple instances of the same type of annotation within the same image and entirely different annotation types are simultaneously included in CE images.

**FIGURE 4:**
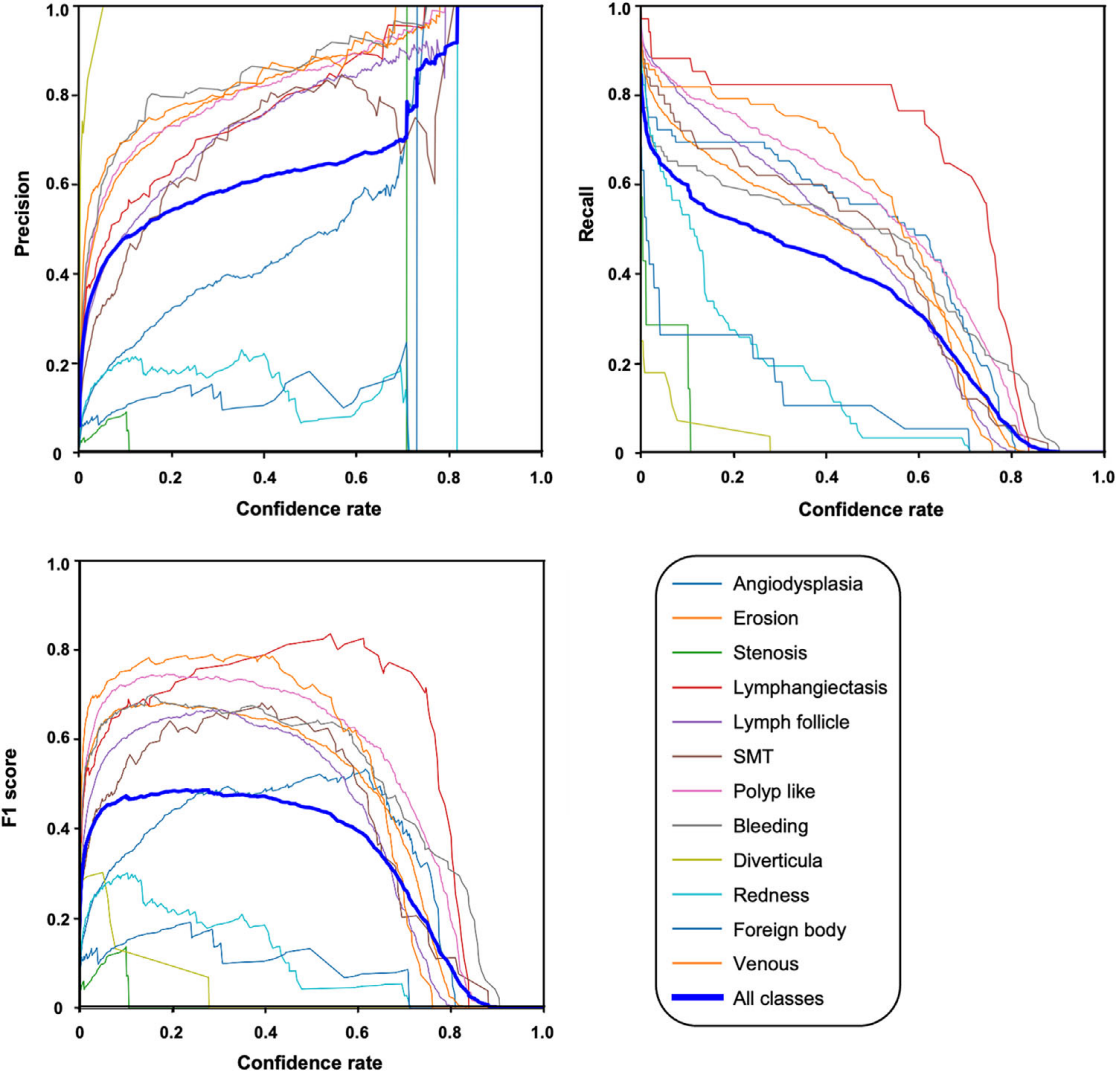
Relationship between Precision, Recall, F1 score, and confidence rate in creating AI model. The point with the highest F1 score had a confidence rate of 0.279.

Since the object detection AI model recognizes specific pathological lesions in an image, it is more appropriate than the classification AI model. The object detection AI model returns the location and confidence rate of the disease lesion. For videos, detection is repeated per frame. Figure 4 shows the relationships among precision, recall, F1‐score, and confidence rate in the model creation. In standard object detection, precision and recall are usually balanced; the cut‐off is often established where the F1‐score is highest. However, when the AI model is used in actual clinical practice, we envision that a specialist will review annotations made by the AI. The F1‐score increased rapidly at confidence rates of ≤0.1, then exhibited a gradual increase.

**FIGURE 5:**
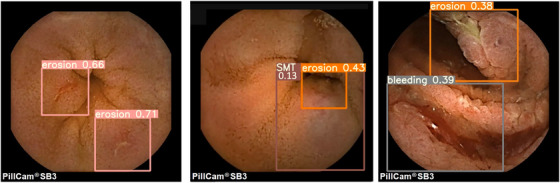
Representative images detected by created artificial intelligence (AI) model. Disease lesions detected by the AI are indicated on a rectangle, along with the confidence rate. The left image detected two different erosions, the middle image detected submucosal tumor (SMT) and erosion at the top, and the right image detected erosion and bleeding (representative detection videos are also included in the dataset).

**FIGURE 6:**
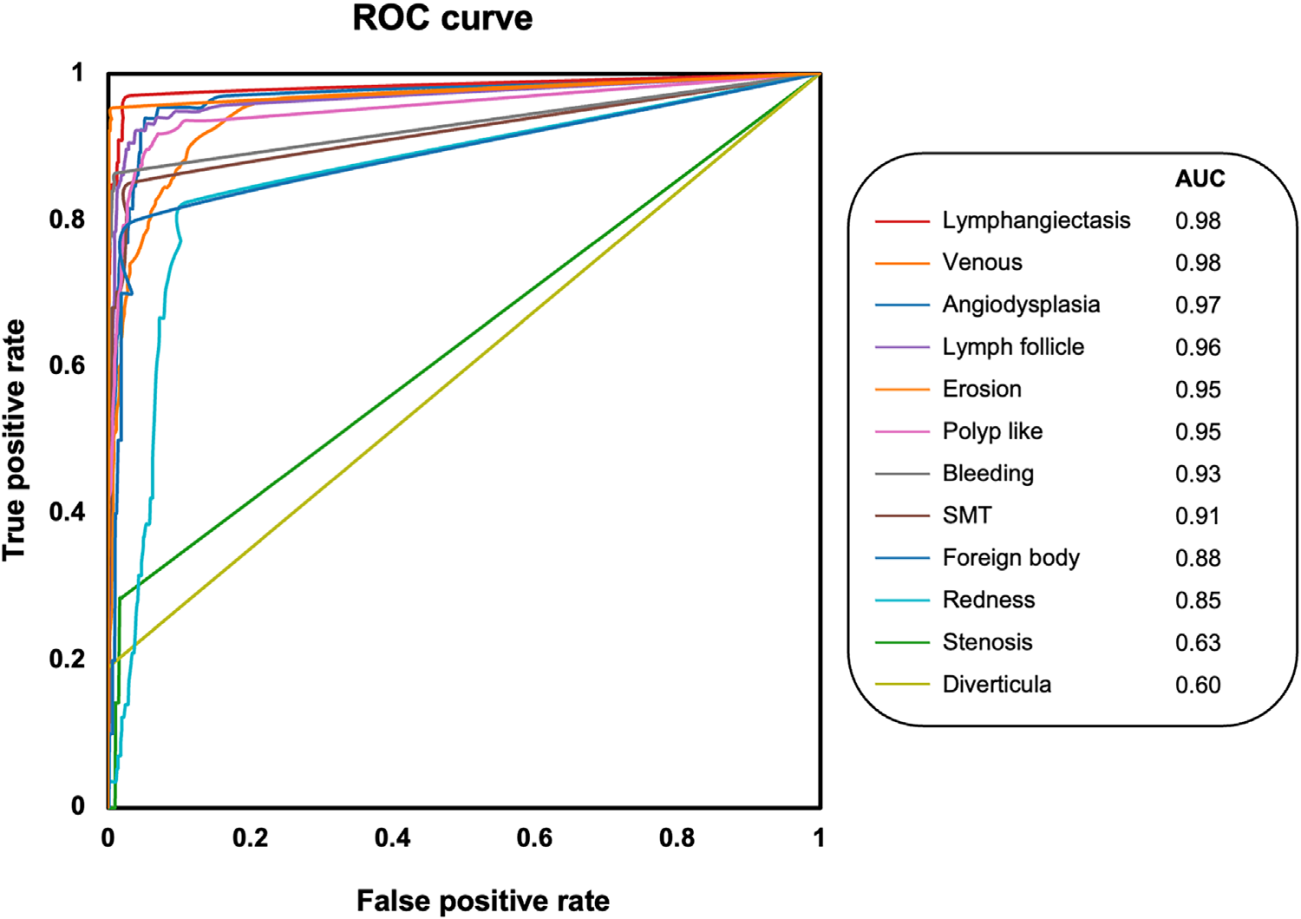
Receiver operating characteristic (ROC) curves for each annotation. The ROC curve is calculated by determining annotations per frame in 1396 test‐validated images. When multiple annotations of the same type are present in the same image, the bounding box with the highest confidence score is utilized in the calculation. The highest area under the ROC curve (AUC) was 0.98 for “venous” and “lymphangiectasis”.

On test validation, there were 1495 annotation types on 1396 test images. Of these, 1375 were detected, resulting in a sensitivity of 91%. Figure 5 shows representative images detected by this AI model, demonstrating multiple representative detections. The left image detected two different erosions, the middle image detected submucosal tumor and erosion at the top, and the right image detected erosion and bleeding (representative detection videos are also included in the Supporting Information). Figure 6 shows the ROC curve for each annotation, with eight of the 12 annotations demonstrating good detection with the area under the curves greater than 0.9. The highest area under the curve was 0.98 for “lymphangiectasia” and “venous.” Table 2 presents the details of the test validation conducted by the created AI model for each annotation type. “Lymphangiectasis,” “venous,” and “angiodysplasia” were well detected by the model. However, the sensitivity for “diverticula” and “stenosis” was found to be low compared to other labels, and many were missed.

**TABLE 2:** Test validation results of the created artificial intelligence (AI) model.

	Venous	Lymphangi ectasis	Lymph follicle	Angio dysplasia	Bleeding	Foreign body	Polyp like	SMT	Erosion	Redness	Stenosis	Diverticula
**True positives**	61	32	107	60	71	9	304	40	484	50	2	4
**True negatives**	1325	1321	1232	1263	1302	1335	994	1305	740	1191	1366	1375
**False positives**	7	42	48	68	13	51	74	44	141	148	23	0
**False negatives**	3	1	9	5	10	1	24	7	31	7	5	17
**Best confidence rate**	0.02	0.01	0.236	0.206	0.015	0.014	0.062	0.011	0.039	0.011	0.041	0.038
**Sensitivity**	0.953	0.97	0.922	0.923	0.877	0.9	0.927	0.851	0.94	0.877	0.286	0.19
**Specificity**	0.995	0.969	0.963	0.949	0.99	0.963	0.931	0.967	0.84	0.889	0.983	1
**Youden Index**	0.948	0.939	0.885	0.872	0.867	0.863	0.858	0.818	0.78	0.767	0.269	0.19

The true positives, true negatives, false positives, best confidence rate, sensitivity, specificity, and Youden Index were calculated by determining annotations per frame in 1,396 test validated images. These values are determined by the best confidence rate (cut‐off value). The sensitivity for “diverticula” was 0.19, and “stenosis” was 0.29, found to be low compared to other labels. SMT, submucosal tumor.

## DISCUSSION

In this study, we created an object detection CE AI model, which classified pathological abnormalities into 12 types of annotations. The ROC analysis shows areas under the curve greater than 0.9 for eight of the 12 annotations. The total sensitivity for the disease lesion was about 91%. More commonly observed annotations had better detection performance, resulting in total higher sensitivity. Nevertheless, AI models currently exhibit lower confidence when detecting “diverticula” and “stenosis.” Since we use open‐source and open our dataset, clinicians can reproduce and use our model.

Previous research has demonstrated that image classification AI models for single lesions in each small bowel CE image can be highly effective.^15^, ^21^ Since the image classification process is based on the whole visual feature of an image, image classification AI models are weak in pointing out multiple types of annotations in the same frame. Previous reports using image classification for CE AI rarely mention instances where different annotation types overlap in the same frame.^22^ As shown in this study, CE images often contain multiple pathological lesions within the same frame. AI using object detection is useful to solve this problem. Object detection is an AI model type that identifies each image's location and type of annotations. For example, if a polyp causes gastrointestinal bleeding with an ulcer, endoscopic hemostasis may be required. Conversely, if the bleeding results from multiple open ulcers, the possibility of Crohn's disease should be considered, and prioritizing appropriate treatment is advisable. The model with multiple annotations is in line with actual clinical practice, and with further advances, it could become a user‐friendly reading aid. Exploratory use of object detection to identify anomalies in CE images has been reported in Single Shot MultiBox Detector and Retinanet.^23^, ^24^ Those reports attempted to detect three to four types of pathological lesions. It is currently uncertain to what extent classification of abnormalities is the most effective reading aid in small bowel CE AI for detecting abnormal images. Ding classified CE images extracted from 6970 individuals into 12 classes, including “normal,” for comprehensive abnormality detection.^15^ When establishing annotations in our AI model, our goal was to classify all important abnormalities for reading; when possible, we sought to combine annotations that rarely need to be distinguished in clinical practice. We divided the pathological lesions into 12 categories, with “stenosis,” “diverticula,” and “redness” being particularly poorly detected by our AI. Regarding “stenosis,” it was challenging to determine whether the intestinal tract was constricted or actively engaged in peristalsis, and concerning “diverticula,” it was difficult to determine whether the image showed a lumen or a diverticulum. Object detection AI cannot account for the previous, and next frames, which we found makes it poor at detecting some disease lesions. For “redness,” it was difficult to draw clear borders; annotation boundaries were easily influenced by subjectivity and often overlapped with other annotation labels. The challenge of annotating such lesions with overlapping classes may contribute to the difficulty in creating high‐level object detection models. To increase the detection rate, more educational images are needed.

The validation of AI models should be carried out using standard images or videos. The creation of standard datasets for CE images will significantly contribute to the development of AI models. For instance, in skin cancer, datasets such as the Melanoma Detection Dataset^25^ and The HAM 10,000 dataset^26^ have been released and are readily accessible. For small bowel CE, several studies have attempted to create large datasets and developed comprehensive AI models for lesion detection. Many of these datasets are no longer available^27^ or may only be obtainable upon request.^28^ Our object detection model and dataset are implemented in the open source and are relatively easy to reproduce, use, and improve.

This study has several limitations. Our dataset was collected from images acquired using the PillCam SB 3. The color and luminosity may vary slightly depending on the manufacturer of the CE system; therefore, the AI model must be modified to detect images from CE devices other than the PillCam SB 3. For example, The Kvasir‐Capsule Dataset^29^ is an existing publicly available large‐scale CE dataset; however, The Kvasir‐Capsule Dataset was acquired using an Olympus EC‐S10 (Olympus, Tokyo, Japan). Thus, it may be challenging to align our dataset with other datasets simply. Our dataset had fewer than 20 cases of most rare diseases, which represents an important limitation. Previous studies have indicated that AI can effectively classify celiac disease^30^ and parasites.^31^ Because there were very few patients with parasites or celiac disease in our hospital, our database is unsuitable for validating AI models trained to detect such diseases. Our test validation, in this case, suggests that our created AI is partially effective at detecting disease lesions and has the potential to serve as a reading aid. However, it has yet to be validated in clinical practice whether this AI leads to a reduction in reading costs or missed findings.

In summary, we make our data and model available to many researchers as a milestone in the technology. The supplement data includes a link to experience our object detection CE AI model on the web‐based Google Colab Notebook. We hope more facilities will collect CE images in the future, supporting the continued improvement and availability of CE AI diagnostic systems to endoscopists.

## CONFLICT OF INTEREST STATEMENT

None.

The link to experience our object detection artificial intelligence and model weights is as follows; https://colab.research.google.com/drive/1mEE5zXq1U9vC01P‐qjxHR2kvxr_3Imz0?usp=sharing

## Supporting information




**Video S1** A sample video detecting submucosal tumor with erosions.

**Video S2** A sample video detecting multiple erosions.

**Video S3** A sample video detecting multiple lymph follicle.

**Video S4** A sample video detecting foreign body (single tablet).

**Video S5** A sample video detecting bleeding.

## Data Availability

Our data are licensed under a Creative Commons Attribution 4.0 International (CC BY 4.0) License. The material is free to copy and redistribute in any medium or format; it can be remixed, transformed, and built upon for any purpose if appropriate credit is given. We have archived the dataset of the project at Kaggle (Google LCC, USA), the world's largest data science online community; DOI: 10.34740/kaggle/ds/1516536.
